# Exploring predictors of instructional resilience during emergency remote teaching in higher education

**DOI:** 10.1186/s41239-021-00278-7

**Published:** 2021-08-09

**Authors:** Joshua Weidlich, Marco Kalz

**Affiliations:** Department of Technology-enhanced Learning, Heidelberg University of Education, Heidelberg, Germany

**Keywords:** Emergency Remote Teaching, Resilience, Higher education, Covid-19, Teaching quality

## Abstract

In 2020, Higher Education institutions were pressed to swiftly implement online-based teaching. Among many challenges associated with this, lecturers in Higher Education needed to promptly and flexibly adapt their teaching to these circumstances. This investigation adopts a resilience framing in order to shed light on which specific challenges were associated with this sudden switch and what helped an international sample of Higher Education lecturers (N = 102) in coping with these challenges. Results suggest that Emergency Remote Teaching was indeed challenging and quality of teaching was impeded but these effects are more nuanced than expected. Lecturers displayed *instructional resilience* by maintaining teaching quality despite difficulties of Emergency Remote Teaching and our exploration of predictors shows that personality factors as well as prior experience may have supported them in this. Our findings may contribute to the emerging literature surrounding Emergency Remote Teaching and contributes a unique resilience perspective to the experiences of Higher Education lecturers.

## Introduction

In 2020, the COVID-19 pandemic necessitated a sudden increase in online-based forms of teaching and learning. Among the many organizations closing facilities to slow the spreading of the virus, higher education institutions quickly moved their classes online and implemented emergency remote teaching (ERT) (Crawford et al., 2020). While some observers were eager to compare the results of this involuntary experiment with face-to-face teaching (Zimmerman, 2020), scholars quickly pointed out that ERT is decidedly different from deliberate and well-designed distance education (Bozkurt & Sharma, 2020; Hodges et al., 2020).

Among the many challenges associated with the switch to ERT, the specific difficulties of quickly and unpreparedly adopting educational practice to the online realm stand out and are still not sufficiently understood. In a recent editorial, Naidu (2021) points to the importance of resilient education systems. Looking instead at individuals as unit-of-analysis, we argue that maintaining teaching quality under these circumstances requires from Higher Education (HE) lecturers not only psychological resilience, but a degree of instructional resilience, a set of attitudes, abilities, and resources that allows HE lecturers to adapt their teaching without sacrificing too much educational quality. Although this concept has not yet been investigated, it appears highly relevant for understanding how HE lecturers were able to cope with the global shift to online teaching ERT and which factors may have provided supports and challenges toward instructional resilience. Investigating this in the context of ERT appears timely and pertinent, as virtually all HE lecturers across the globe have been confronted with the challenges of suddenly adapting their teaching (Bozkurt et al., 2020), thus eliciting some degree of instructional resilience.

The present study reports on the results of a survey-based data collection exploring the experiences of HE lecturers in maintaining teaching quality during ERT, the construct of instructional resilience as a result of these experiences, as well as different predictors that may have contributed to or deterred from demonstrating instructional resilience during ERT. This research aims to understand the experiences of HE lecturers during ERT, the challenges they encountered, and their ability to face these challenges. As the consequences of ERT will be felt, researched, and discussed for years to come, this study aims to contribute a piece of the puzzle of what contributed to educational success and what we can do to improve instructional resilience in HE lecturers.

## Literature review

Although there is not yet any research on instructional resilience per se, we can turn first to the very large literature on the general construct of resilience and to the smaller but substantial literature on teacher resilience to get a handle on the central construct of this research. After this, we will show how instructional resilience differs from these broader kinds of resilience and briefly review different types of potential risk and protective factors.

### What is resilience?

General psychological resilience is the ability of an individual to cope with a crisis. It is, thus, considered an integral component of mental health in general (Davydov et al., 2010), important for child development (Garmezy, 1991), but is also, for example, frequently investigated with respect to stressful and/or high-risk occupations (De Terte & Stephens, 2014). Importantly, there is an emerging consensus that psychological resilience is not a fixed trait or an innate set of non-amenable attributes. Instead, it is seen as a dynamic process, influenced as much by contextual factors, attitudes, and availability of resources as well as individual differences in personality (Gu & Day, 2007; Sattler et al., 2014; Sudom et al., 2014). As such, the empirical study of resilience has explanatory power because we can learn about the “why”, “when”, and “how” of individual’s positive adaption in the face of adversity. This opens up the possibility of improving said contextual factors, attitudes, and availability of resources to deliberately promote resilience in different contexts.

As an important domain-specific conceptualization of resilience, we can turn to resilience in education. Within education, the most widely researched context is K-12 education, or what is simply called teacher resilience. As the difficulties of early-career lecturers are well documented in the literature (Tait, 2008) and teacher attrition is an issue in many countries (Scheopner, 2010), here too, research has focused on what helps lecturers sustain and thrive in order to deliver quality education (Gu & Day, 2007; Mansfield et al., 2012, 2014). A review based on 50 studies by Beltman et al., (2011) identifies risk factors as well as protective factors, which can be individual and contextual, respectively. Individual risk factors include negative self-beliefs or difficulty in asking for help, whereas contextual risk factors include difficulties in class management or heavy workloads. On the other hand, individual protective factors include strong intrinsic motivation and self-efficacy, whereas contextual protective factors include strong leadership and positive student–teacher relationships.

As another educational sector increasingly known for its occupational stress, resilience in higher education lecturers is becoming a topic of interest (Gillespie et al., 2001; Helker et al., 2018; Watts & Robertson, 2011). As some of the biggest academic stressors, competing obligations, conflicting goals, and job insecurities in academia emerge time and again in the literature (Cretchley et al., 2013; May et al., 2013; Navarro & Màs, 2010). Taking an individual perspective, Helker et al. (2018) identify four personal dimensions that characterize a resilient university teacher: a profession-related dimension (e.g. accepts imperfection), a social dimension (e.g. good private network), a motivational dimension (e.g. intrinsically motivated), and an emotional dimension (e.g. problem distance).

### Instructional resilience during ERT

ERT due to the Covid-19 pandemic has brought about another major stressor for HE lecturers but also provides us with a unique situation to understand a specific type of resilience among HE lecturers, what we call instructional resilience. Although the digital transformation of the HE sector has been an important topic of discussion and research for years prior (e.g., Bond et al., 2018; Jensen, 2019), the sudden switch ERT has brought to light the deficiencies and lack of preparedness of a high-inertia system like HE (Bozkurt et al., 2020; Naidu, 2020; Zawacki-Richter, 2020). However, despite hardships and struggles, interim evaluations must concede that ERT “worked” to some degree (Bonk, 2020; Kerres, 2020; Lederman, 2020). That is, although there were certainly huge disparities between institutions and countries (e.g. Khlaif et al., 2021), it appears that in many cases the switch to ERT occurred swiftly and was sustained over the course of weeks and months, in order to provide continued education to university students. Although the jury is still out regarding the net learning effects of this involuntary experiment, we can preliminarily wager that ERT has not been a failure (Bawa, 2020; Gonzalez et al., 2020). This preliminary result is noteworthy as university lecturers’ readiness for online-based learning is highly heterogenous (Scherer et al., 2021) and many HE lecturers have not felt prepared nor confident in delivering online education (Watermeyer et al., 2020).

As a key assumption of this study, we suggest that the sustained provision of education in HE is, at least partly, due to instructional resilience of HE lecturers. In other words, the extent to which ERT was successful in providing quality education can be attributed to the ability of HE lecturers to adapt their teaching to these new circumstances. This ability to sustain or even increase efforts in the face of adversity clearly connects our construct to the general notion of psychological resilience. Yet, it goes beyond broad resilience by more specifically capturing the pedagogical or educational nature of this adaptation. HE lecturers are instructionally resilient not merely by ‘pushing through adversity’, but by using prior knowledge, personal experience, and different resources to make adaptions in their teaching practices in light of this adversity. As a research opportunity, the global and comprehensive nature of the switch to ERT makes it unique, as HE institution across the globe made this switch and likely all HE lecturers had to adapt to some extent. Our central assumption is that the degree to which teaching quality was maintained despite these challenges is a function of the instructional resilience of HE lecturers. In line with work on teacher resilience, we define a reference point for the display of instructional resilience (Schwarze & Wosnitza, 2018). In our case, this is the relative upholding of teaching quality during ERT, compared to before ERT.

### Predictors of instructional resilience

Regarding factors that may predict instructional resilience, we derive a typology of potential factors based on the literature of teacher resilience. Following Beltman et al., (2011), we find it plausible to distinguish between individual (i.e. personal) attributes and contextual (i.e. work-related) factors. Among individual attributes, we further distinguish between general aspects of personality and relevant prior experience. Besides basic demographic information, this leaves us with three subsets of potential predictors of instructional resilience, (1) personality attributes, (2) prior experience, and (3) contextual factors.

Personality attributes are basic patterns of thinking, feeling, and behaving within an individual (Roberts et al., 2014). The most widely used and researched model of personality is the Big Five model (Costa & McCrae, 1992). This is a good starting point to understand personality-based predictors of instructional resilience as the Big Five captures relatively broad and enduring set of individual differences in personality. In addition, and somewhat more specific, it appears plausible that psychological resilience is an important personality attribute predicting instructional resilience. Viewing ERT as a crisis within the HE system that has added novel and highly salient stressors to the environment, we suggest that general adaptive coping (i.e. resilience) facilitates specific teaching-related coping (i.e. instructional resilience). Finally, we include causality orientation as potential personality predictors. These are motivational constructs embedded in personality (Deci & Ryan, 1985). Individuals high on autonomous causality orientation are more motivated, seek challenges, and perceive high degrees of agency whereas impersonal causality entails low self-efficacy, anxiety, and amotivation (Ryan & Connell, 1989). Viewing ERT either as a challenge that can be approached or an unavoidable calamity may impact a HE lecturers’ ability and willingness to adapt teaching practice and, thus, instructional resilience.

As a second set of personal attributes that is more specific to the pedagogic nature of instructional resilience, we suggest relevant prior experience. Alongside Scherer et al. (2021) who found that prior experience predicted readiness for online learning, we suggest that HE lecturers with prior experience in TEL/DE can draw on knowledge, experience, and resources to adapt their teaching to ERT and, thus, demonstrate instructional resilience. We consider four categories of prior experience, (1) having engaged with professional development (formal and non-formal) with respect TEL/DE, (2) general self-rated ability to use technology for teaching purposes, (3) working in the field of TEL/DE, and (4) working at an institution providing distance education or online learning. We hypothesize that all of these types of prior experience may be protective factors with respect to instructional resilience during ERT.

Outside from individual attributes, we can also consider contextual factors that are work-related. Drawing on research from occupational resilience (Greifer, 2005; Hartmann et al., 2020), we suggest that differences in work environments may predict instructional resilience. To represent this set of predictors, we chose the degree to which HE lecturers perceived organizational support, social support, technical support as well as the workload.

## Research questions

Currently, there remains a large gap in research regarding our understanding of HE teacher’s challenges and difficulties during ERT. Thus, before investigating instructional resilience as a specific type of adaption, we first need to get a comprehensive picture of the experiences of HE lecturers as they made the switch to ERT in 2020. Thus, our first research question is:

### RQ1:

What are the experiences of HE lecturers in maintaining teaching quality during ERT in HE?

To answer this question, we assess the perceived quality of teaching during ERT, the perceived challenge of teaching during ERT, as well as aspects of learning design that were considered particularly challenging/easy to implement during ERT. With respect to this research question, we will also look at contextual factors (e.g. workload, technical support, and social support) to arrive at a deeper understanding of the challenges and supports perceived by HE lecturers. After getting an understanding of the degree of difficulty that HE lecturers perceived during ERT, our second research question then more deeply considers what may have contributed to lecturers’ sustaining efforts and adapting their teaching, i.e. instructional resilience. Learning about this can give us valuable insights about what did or did not make ERT “work”. Thus, our second research question is:

### RQ2:

Which factors contribute to demonstrating instructional resilience during ERT in HE?

Regarding factors that may predict instructional resilience, we base our analysis on the three sets of potential predictors based on the literature of resilience: personality attributes, prior experience, and contextual factors. With the addition of control variables (age and gender) we therefore have a total of four classes of potential predictors, which themselves can be further distinguished into constructs and variables representing these predictor classes. As an exploratory work into the novel concept of instructional resilience, we attempt to sample a relatively broad array of constructs and variables to represent these predictor classes (see “Measures”).

## Method

### Data collection

To collect data about teaching experiences, instructional resilience, and potential predictors, we developed a questionnaire using Limesurvey (https://www.limesurvey.org). It included a total of 75 items, which pre-testing suggested take approx. 15 min to complete. There was no reward or incentive associated with participation. We started distributing our questionnaire through a number of channels on November 12th, 2020. Among these channels were our institution’s mailing list, Twitter, Facebook groups, LinkedIn and ResearchGate. For example, we searched for international Facebook and LinkedIn groups focused on teaching in Higher Education, joined them if possible, and informed the moderators about our data collection and asked if we could recruit participants from their group. Our recruitment via ResearchGate was done by using the “Questions” feature. Within this feature we used both the “ask a technical question” and “start a discussion” functionality. Still, we quickly noted that data collection was slow despite our efforts (less than 40 full responses in November) and we suspected a type of survey fatigue to be the issue (Porter et al., 2004), as 2020 was not only generally stressful for HE lecturers but we also noticed an increase of data collections relating to Covid-19-related topics. For this reason, we decided to decrease our efforts of data collection for the time being and wait until after December. Then, in January we again advertised for our survey using the same channels and found responses to come in faster. Data collection remained open until February 7th, 2021, when we surpassed 100 full responses. Response statistics indicated a mean response time of 15.8 min (SD = 6.3). Participants whose country were not affected by the Covid-19 pandemic and participants whose institutions did not switch to ERT were excluded at the beginning of the questionnaire.

### Sample

Our sample consists of N = 102 full responses. Of these, 64 participants (62.7%) were women, 35 (34.3%) were men, one person (1%) indicated “diverse”, whereas two persons (2%) chose to give no response. Regarding age, most participants were in the age categories of 26–35 (25.5%), 36–45 (29.4%), and 46–55 (23.5%) years old. We recruited very few participants younger than 26 (5.9%), some above 55, 56–65 (13.7%), and only two that were older than 65 years old (2%). Most HE lecturers work in the field of Education (34.7%). The second most indicated areas of teaching are Social Science excl. Education with 14.3% and Humanities & Liberal Arts with 12.2%. Natural Sciences and Engineering were somewhat less represented with 7.1% each. 12 participants responded with “other”, indicating such diverse fields as Health Sciences, Accounting, Design, Computer Science, and Maritime Education. The HE lecturers in our sample come from 27 different countries, with Germany being the most represented (24%), followed by UK (5.3%), US (4.2%) and Netherlands (3%). Countries represented by 1–3 respondents include, for example, Austria, Bulgaria, India, Malaysia, Norway, Romania, Oman, Switzerland, Turkey, South Africa, and Ukraine.

Of our total sample, exactly half (n = 51) indicate an affiliation with the broader area of Technology-enhanced Learning (TEL) or Distance Education (DE). More than half of our sample (54.9%) are affiliated with brick-and-mortar institution that have provided little or no online offerings before 2020. About one-third (29.4%) work at a hybrid institution, with the remaining 15.7% working at a fully online institution. Chi-square test of contingency indicates that respondents working in the broader field or TEL or DE are more likely to be affiliated with an institution providing some type of online education, *x*^2^(2, *N* = 102) = 6.02, *p* = .049. In terms of teaching experience, about one-third of participants indicated the category with the lowest duration of experience, 1–5 years (34.7%). Second, with one-fifth of responses were 11–15 years (21.4%). The remaining categories were endorsed as follows: 6–10 years (16.3%), 16–20 years (12.2%), and more than 20 years (15.3%). Regarding teaching load prior to ERT, respondents answered as follows: 1–3 h/week (14.7%), 4–6 h/week (24.5%), 7–9 h/week (16.7%), 10–12 h/week (17.6%), 13–15 h/week (6.9%), and more than 15 h/week (19.6%). Finally, most respondents (76.5%) indicate having engaged in deliberate efforts to improve knowledge about TEL or DE. This remained true even while looking at only the half of sample not affiliated with this research field (58.8%).

### Measures

We collected data using validated measures (see Table 1), the only exception being our measure of instructional resilience, as this construct has not been investigated in the literature (see “Results” section for validity evidence). Aside from the General Causality Orientation Scale (GCOS), which was measured on a 7-point Likert scale, all remaining scales were measured on a 5-point Likert scale ranging from “1—strongly disagree” to “5—strongly agree”. Most of our measures showed adequate internal consistence (> .7) as measured via Cronbach’s Alpha. An exception to this is the Big Five personality inventory used here. In an attempt to not burden our respondents excessively and keep the survey as short as possible, we decided to use the BFI-2-SX (Soto & John, 2017), which assesses each personality dimension with only three items. Of course, this brings about psychometric limitations, the results of which can be found in the relatively low values of internal consistency. Regarding the dimension of Openness, we decided to exclude this subscale from further analyses, as an internal consistency < .5 appeared indefensible. In order to assess HE lecturers’ perceived ability to use technology for teaching, we used a short-form measure (Schmid et al., 2020) from the TPACK framework (Mishra & Koehler, 2006). Technological-pedagogical content knowledge (TPACK) of HE lecturers was assessed twice, once with respect to HE lecturers’ knowledge prior to ERT as well as after ERT. Respondents were asked to indicate identical values if they did not perceive their ability to use technology for teaching to have changed due to ERT. The numbers in parentheses indicate the final number of items used for analyses, in the few rare instances where exclusion of items was warranted due to lack of unidimensional factor structure. Not listed in Table 1, we also asked respondents to indicate the perceived quality of teaching during ERT in percent, relative to their teaching practice before ERT. With this variable, a response of 100% means that a HE teacher did not perceive his/her teaching to suffer at all during ERT. Finally, we also asked about the perceived challenge of teaching during ERT in general, which participants could answer on a 7-point scale ranging from “1—very easy” to “7—very challenging” (Appendix).

**Table 1:** Psychometric measures used in this study

	#items	Example item	Cronbach’s alpha	References
Instructional resilience	6	“Despite the challenges of remote teaching, I was able to teach my students effectively”	.88	New
General resilience	6	“I usually come through difficult times with little trouble”	.85	Brief Resilience Scale Smith et al. (2008)
TPACK before/after ERT	4	“I was/am able to choose technologies enhance the content for a lesson”	.92	TPACK.xs Schmid et al. (2020)
Extraversion	3	“… is dominant, acts as a leader”	.64	BFI-2-SX Soto and John (2017)
Agreeableness	3 (2)	“… is compassionate, has a soft heart”	.52	BFI-2-SX Soto and John (2017)
Conscientiousness	3	“… has difficulties getting started in tasks” (r)	.55	BFI-2-SX Soto and John (2017)
Neuroticism	3	“… is emotionally stable, not easily upset”	.76	BFI-2-SX Soto and John (2017)
Openness	3	“… has little interest in abstract ideas” (r)	.46	BFI-2-SX Soto and John (2017)
Impersonal causality	6	New position: “What if I can’t live up to the new responsibilities?”	.76	GCOS Deci and Ryan (1985)
Autonomous causality	6	New position: “I wonder if the new work will be interesting?”	.73	GCOS Deci and Ryan (1985)
Workload	5 (3)	“There seemed to be too much work to get though here”	.74	Workplace Climate Kirby et al. (2003)
Organiz. support	8	“My organization values my contributions to its well-being”	.9	POS Eisenberger et al. (1986)
Technic. support	7	“Technical support provided timely answers”	.94	TSCSS GuideStar Research (2005)
Social support	6 (4)	“If needed, can you talk with your friends about work-related problems?	.82	QPS Nordic Ørhede et al. (2000)

### Data analysis

To investigate the experiences of HE lecturers during ERT, we first analyzed if and how they perceived their teaching quality to have changed with a one-sample t-Test, with 100% being considered the reference value (i.e. no change in teaching quality). The existence of an association between degree of subjective challenge during ERT and teaching quality was assessed using Spearman’s rho. Differences in teaching load were analyzed using a one-sample t-Test, with 0 being the reference value (i.e. no change in teaching load. To understand learning design experiences during ERT, we descriptively compared which design features HE lecturers felt they were able to implement well versus unable to implement well, identifying those features most (un)popular and those most (un)ambiguous. To understand if HE lecturers found themselves generally better able to use technology for teaching purposes as a result of ERT, we compared their ratings (before and after ERT) with a paired-sample t-Test. Associations between workplace challenges and supports were investigated via correlation analysis (Pearson) and open-ended questions about supports and challenges were synthesized via thematic analysis (Braun & Clarke, 2006), in which response were clustered according to larger themes and only themes with two or more representative responses were included for reporting.

To investigate factors associated with Instructional Resilience during ERT, we first analyzed our scale in terms of its factor structure using Exploratory Factor Analysis and internal consistency using Cronbach’s Alpha. Evidence of convergence validity was obtained by inspecting correlations (Pearson) with theoretically related variables. To explore different predictors of Instructional Resilience, we used a hierarchical linear regression approach, where we built four regression model of increasing complexity, starting with control variables in block 1 and then adding three more sets of predictors, personality attributes, relevant experience, and institutional factors. Predictive ability of these models was assessed via *R*^2^ for individual models and change of *R*^2^ (Δ*R*^2^) between models.

## Results

As some nominal predictor variables had low cell counts, we collapsed small cells into larger cells while attempting to retain the distinctions within these variables as much as possible during data preparation. For example, there were very few participants in our ‘26 and under’ age category (n = 6) so that we collapsed this age category into the next higher category, creating a new ‘younger than 36’ category. This was also done for the opposite side of the age distribution, creating a new ‘older than 55’ category. Similarly, because of relatively few HE lecturers from fully-online universities, we also collapsed the three institution types into two categories, ‘Brick-and-Mortar’ and ‘Hybrid or Fully-online’.

### Results for RQ1

#### What are the experiences of HE lecturers in maintaining teaching quality during ERT?

##### Teaching quality, challenge, and teaching load

**Fig. 1:**
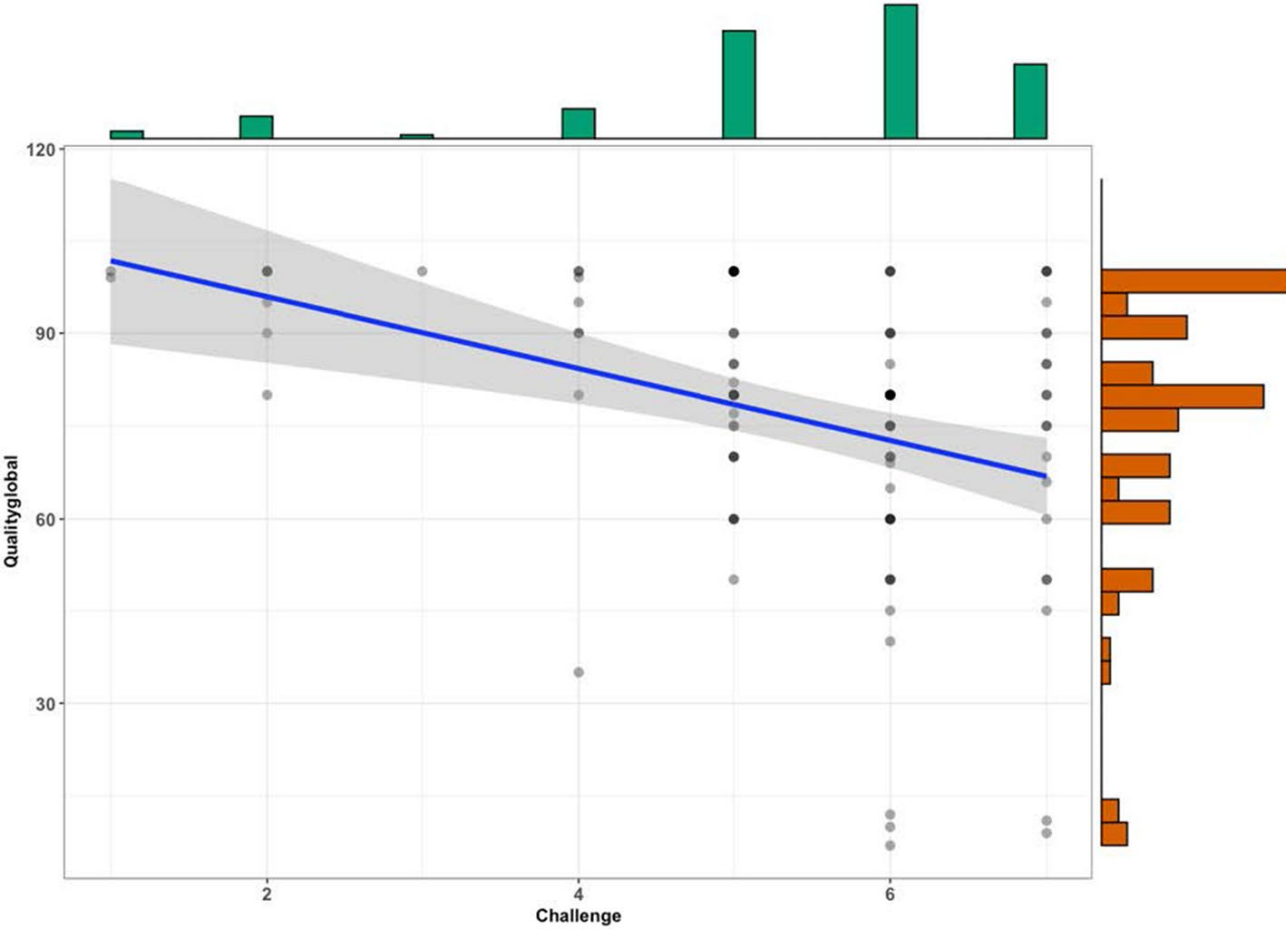
Bivariate relationship of teaching quality and perceived challenge during ERT

HE lecturers found their perceived teaching quality to have suffered during ERT, with a mean of responses around 76% (SD = 22.2), indicating a one-quarter drop in teaching quality, compared to their teaching prior to ERT. One-sample t-Test (H^0^ = 100) indicates that this is a statistically significant decrease from the reference value of 100, *t*(101) = − 10.8, *p* < .001. Almost 15% of HE lecturers indicate their teaching quality reduced by 50% or more. On the other hand, and perhaps surprisingly, a substantial number HE lecturers (19.6%) responded with 100%, indicating no reduction of teaching quality due to ERT. HE lecturers also found ERT to be challenging, as indicated by high mean values on our perceived challenge item, *M* = 5.4 (SD = 1.4). Perceived challenge and quality correlate negatively *r* = − .37, *p* < .001 (Spearman’s rho), indicating that HE lecturers who perceived ERT to be challenging times also reported lower teaching quality (see Fig. 1). There were no differences in quality of teaching and perceived challenge between HE lecturers along the lines of gender, age groups, area of teaching, years of teaching, type of institution, work in the field of TEL/DE, or professional development.

Besides teaching quality and perceived challenge, we also asked HE lecturers to indicate their average teaching load including preparation, office hours, and supervision before ERT and during ERT. The change in teaching load was calculated by subtracting teaching load prior to ERT from teaching load during ERT, the results of which can be seen in Table 2. Almost half of our sample of HE lecturers indicate no change in teaching load induced by ERT, while almost 15% indicate a decrease in teaching load and more than 37% indicate an increase in teaching load. One-sample t-Test (H^0^ = 0) indicates that this is a statistically significant increase compared to the reference level 0, *t*(101) = 2.07, *p* < .041. There were no differences in teaching load change between HE lecturers along the lines of gender, age groups, area of teaching, years of teaching, type of institution, work in the field of TEL/DE, or professional development.

**Table 2:** Teaching load change during ERT

Difference	Counts	% of total
− 5	1	1%
− 4	1	1%
− 3 (much less)	1	1%
− 2	5	4.9%
− 1	7	6.9%
0 (same)	49	48%
1	23	22.5%
2	12	11.8%
3 (much more)	3	2.9%

##### Learning design and teaching with technology

**Fig. 2:**
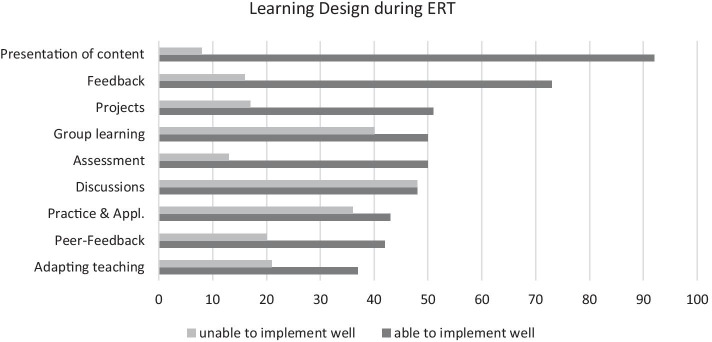
Perceived (in-)ability of HE lecturers to implement learning design features during ERT

In order to get a better understand about how ERT may have impacted learning design, we asked our participants to mark features of learning design they found themselves ‘able to implement well’ versus ‘unable to implement well’ during ERT (see Fig. 2). HE lecturers found that they were well able to implement ‘Presentation of content’ during ERT, with over 90% of respondents indicating their ability in this dimension of learning design and less than 8% indicating deficiencies. This yields a ratio of 11.5, making ‘Presentation of content’ the most unambiguous easy-to-implement learning design feature. Second is ‘Feedback’ with more than 70% endorsing their ability to implement this and a ratio of 4.6 ability/inability. Adapting teaching to students’ knowledge was the least endorsed learning design dimension, showing that less than 40% of participants were able to implement this well during ERT. Similarly, difficult-to-implement were ‘Peer-feedback’ as well as ‘Practice & Application’, with slightly more than 40% indicating ability to implement these features. However, with a ratio of 1.2 inability/ability, ‘Practice & Application’ appears to be more ambiguous in terms of its difficulty of implementation during ERT than ‘Peer-feedback’, which appears more clearly to pose problems (2.1 inability/ability). ‘Discussions’ are the most ambiguous learning design features, with the same amount of HE lecturers indicating ability as inability to implement this, yielding a ratio of exactly 1 inability/ability.

HE lecturers responded to the ‘other’ option in the ‘unable to implement well’—category with 21 free-text responses, among them, e.g. “field trips”, “lab work”, “social dimension of learning”, “workshops”, “non-verbal aspects of interaction”, “experiential learning”, “flexibility in learning design” and “student engagement”. There were no responses to open-ended responses in the ‘able to implement well’—category.

Regarding HE lecturers’ general ability to use technology for teaching, we find significant changes in their reports. Looking back to before ERT, HE lecturers reported a mean of 3.67 (SD = .93), whereas after ERT of summer 2020, they reported a mean of 4.18 (SD = .7). Paired samples t-Test yields a statistically significant difference, *t*(101) = 6.93, *p* < .001, Cohen’s d = 0.68, indicating that the experiences during ERT resulted in substantially higher estimation of their abilities to use technology for teaching purposes.

##### Workplace challenges and supports

In terms of workplace challenges and supports, HE lecturers in our sample reported relatively high degrees of organizational support (*M* = 3.2, SD = .85) and social support at work (*M* = 3.5, SD = .92), a rather high workload (*M* = 3.4, SD = .76), as well as satisfaction with technical support (*M* = 3.5, SD = .98). Analysis of bivariate correlations (see Table 3) show that different types of support are positively associated, whereas workload is negatively associated with organizational and social support but not technical support. Notably, none of these workplace factors are significantly associated with the perceived challenge of ERT for HE lecturers.

**Table 3:** Zero-order correlations (Spearman’s rho)

	(1)	(2)	(3)	(4)	(5)
Organizational support (1)	–				
Social support (2)	.38***	–			
Technical support (3)	.39***	.42***	–		
Workload (4)	− .27**	− .23*	− .07	–	
Perceived challenge (5)	− .17	− .09	− .10	.13	–

*p < .05, **p < .005, ***p < .001

Open ended questions asking about which factors in their job and institution they found particularly helpful and supportive (unhelpful and challenging) during ERT yielded the following themes: Online teaching expertise within the institution, well-working technical infrastructure, helpful and quick technical support, supportive climate and policy, general availability of digital technology. Themes for unhelpful factors were: lack of recognition, insufficient strategy and support, constantly shifting technologies and requirements, lack of transparency and communication, too many regulations with respect to data protection, overburdening of staff, lack of online teaching expertise, disregard for increased workload, and technological issues.

Notably, we found differences in terms of organizational support and social support between brick-and-mortar institutions and those that teach hybrid or fully online. HE lecturers at hybrid or fully online institutions report higher degrees (*M* = 3.43, *SD* = .81) of organizational support than at brick-and-mortar institutions (*M* = 3, *SD* = .84), *t*(100) = 2.59, *p* = .01. The same holds true for social support (*M* = 3.35, *SD* = .89 and *M* = 3.72, *SD* = .94, respectively), *t*(100) = 2.03, *p* = .04. No such differences were found for technical support or workload. Also, HE lecturers working in the field of TEL/DE report higher organizational support during ERT, *t*(100) = 2.42, *p* = .02. There were no further differences in workplace challenges and supports along the lines of gender, age groups, area of teaching, years of teaching, or professional development.

### Results for RQ2

#### Which factors contribute to experiencing instructional resilience during ERT in HE?

Our new Instructional Resilience variable was assessed in terms of factor structure and internal consistency. Bartlett’s Test of Sphericity and KMO measure of sampling adequacy suggested suitability for factor analysis, *X*^2^ = 349.44, *p* < .001, KMO’s = .76–.85, respectively. Exploratory Factor Analysis using Maximum Likelihood extraction, Oblimin rotation, based on an Eigenvalue of 1 suggested a one-factor solution with factor loadings ranging from .58 to .9, accounting for a total variance of 55.8%. Internal consistency using Cronbach’s alpha was high at .88. In addition, convergence validity was assessed by analyzing correlations between Instructional Resilience and the perceived quality of teaching during ERT as well as perceived challenge during ERT. We expect these measures to converge such that Instructional Resilience strongly positively correlates with the perceived quality of teaching during ERT and somewhat weaker negatively correlates with perceived challenge during ERT. We find evidence to support convergence validity, as Instructional Resilience correlated with teaching quality as expected, *r* = .50, *p* < .001 and with perceived challenge as expected, *r* = − .38, *p* < .001. Taken together, we provide evidence for convergence validity, construct validity and internal consistency of our Instructional Resilience variable (Table 4).

**Table 4:** Items, factor loadings, and uniqueness for instructional resilience construct

	Item content	Factor loading	Uniqueness
IR_1	My teaching quality did not suffer from the abrupt change to emergency remote teaching	.90	.19
IR_2	Despite the switch to remote teaching, I was able to conduct my teaching with the usual quality	.89	.2
IR_3	My teaching suffered from the emergency remote teaching of last semester. (r)	.62	.61
IR_4	I estimate that my students learned less in this past semester of emergency remote teaching than in the semesters prior. (r)	.58	.62
IR_5	Despite the challenges of the remote teaching semester, I was able to teach my students effectively	.71	.5
IR_6	I was able to adapt my teaching to the conditions of remote teaching while upholding my usual standards of quality	.72	.49

To explore predictors of instructional resilience during ERT, we conducted a hierarchical linear regression in four stages, adding one class of predictors in every stage. As outlined earlier, these are (1) control variables, (2) personality attributes, (3) relevant experience, and (4) institutional factors. Assumptions were met for Shapiro–Wilk test of normality (S–W = .99, *p* = .53), Goldfeldt–Quandt test of heteroskedasticity (G–Q = 1.51, *p* = .14), Durbin–Watson test for autocorrelation (D–W = 1.90, *p* = .56). Variance Inflation Factors ranging from 1.01 to 1.44 suggest no issues of collinearity.

Regressing instructional resilience on control variables in block 1 show a significant difference between youngest HE lecturers and oldest HE lecturers (see Table 5). The direction of this association is such that older HE lecturers report higher degrees of instructional resilience than younger HE lecturers. Further age comparisons as well as gender revealed no significant associations. With an *R*^2^ of .05, this model explains around 5% of variance in instructional resilience.

**Table 5:** Results of hierarchical regression with four blocks of predictors and instructional resilience as dependent variable

		Model 1			Model 2			Model 3				Model 4		
		*β*	*t*	*p*	*β*	*t*	*p*	*β*	*t*	*p*		*β*	*t*	*p*
	Intercept	3.21	17.69	< .001	2.22	2.10	.04	2.00	1.80	.07		2.44	2.01	.04
Block 1: control variables	Age													
	< 46–< 36 years	0.23	1.03	.31	0.13	0.62	.54	0.11	0.53	.60		0.12	0.55	.58
	< 56–< 36 years	0.23	0.98	.33	0.05	0.23	.82	− 0.01	− 0.50	.96		0.00	0.01	.99
	> 56–< 36 years	**0.61**	2.28*	**.03**	0.36	1.35	.18	0.29	1.13	.26		0.25	0.95	.34
	Gender													
	Female–male	− 0.01	− 0.05	.96	− 0.18	− 0.99	.33	− 0.18	− 1.04	.30		− 0.21	− 1.19	.24
Block 2: personality attributes	General resilience				− 0.05	− 0.38	.33	− 0.03	− 0.20	.84		− 0.01	− 0.08	.94
	BF extraversion				− 0.15	− 1.27	.71	− 0.17	− 1.51	.14		− 0.13	− 1.10	.28
	BF agreeableness				0.12	1.01	.21	0.08	0.63	.53		0.04	0.31	.76
	BF conscientiousness				**0.37**	3.19**	**.002**	**0.28**	2.50*	**.01**		**0.28**	2.38*	**.02**
	BF neuroticism				0.07	0.12	.55	0.08	0.67	.51		0.09	0.71	.48
	Impersonal causality				**− 0.25**	− 2.46*	**.016**	**− 0.22**	− 2.27*	**.03**		**− 0.21**	− 2.08*	**.04**
	Autonomous causality				0.13	1.37	.18	0.13	1.48	.14		0.15	1.55	.13
Block 3: relevant experience	Prof. development													
	No–yes							− 0.4	− 1.88	.06		− 0.45	− 1.96	.05
	Teaching with technol							**0.19**	2.04*	**.04**		**0.20**	− 2.03*	**.04**
	TEL/DE related field													
	No–yes							0.00	0.04	.97		0.00	0.05	.96
	Institution type													
	(some) online–offline							0.01	0.08	.94		− 0.00	− 0.01	.99
Block 4: institutional factors	Organizational support											− 0.12	− 1.03	.31
	Social support											0.08	0.71	.48
	Technical support											0.05	0.50	.62
	Workload											− 0.12	− 0.97	.33
	Model fit	*R* = .23			*R* = .49			*R* = .58				*R* = .60		
		*R*^2^ = .05			*R*^2^ = .24			*R*^2^ = .34				*R*^2^ = .36		
	Model comparison	Δ*R*^2^ = .18, F(7, 87) = 3.00**, *p* = .007					Δ*R*^2^ = .09, F(4, 83) = 3.04**, *p* = .022				Δ*R*^2^ = .02, F(3, 80) = 0.62, *p* = .65			

Significant coefficients are highlighted in bold

*p < .05, **p < .005

Adding the second block in model 2 substantially and significantly increases prediction of instructional variables with an *R*^2^ = .24, thus explaining almost one-fourth of variance. This increase is statistically significant, Δ*R*^2^ = .18, F(7, 87) = 3.00, *p* = .007. In this model, we find that more conscientious HE lecturers report higher degrees of instructional resilience, whereas those with an impersonal causality orientation report lower degrees of instructional resilience. Notably, general psychological resilience, autonomous causality orientation, and neuroticism, all theoretically plausible predictors of instructional resilience, show no significant association. Also, with the addition of these personality attributes, the statistically significant association of age in model 1 disappears.

With the addition of the third block of predictors in model 3, prediction of instructional resilience again significantly increases, Δ*R*^2^ = .09, F(4, 83) = 3.04, *p* = .022. With an *R*^2^ of .34, this model explains more than one-third of variance in our dependent variable. Conscientiousness and impersonal causality remain significant predictors, although—expectedly—less strongly so. Of the predictors in this block, only self-rated ability to teach with technology is statistically significantly associated with instructional resilience, suggesting that HE lecturers reporting higher degrees of this ability before ERT also demonstrated more instructional resilience during ERT. Notably, working in the field of TEL/DE and working at a HE institution with online offerings before ERT were not significant predictors of instructional resilience. Descriptively, although not statistically significant, having engaged in professional development regarding teaching with technology appears to have protective effects for some HE lecturers but less so for others. With *B* = − .45 this is the highest estimate in model 4 but due to large variance of this relationship, the 95% confidence interval crosses zero [− 0.90; 0.01], making it statistically non-significant.

Finally, in our fourth model, we only find minor and non-significant improvements in prediction, Δ*R*^2^ = .02, F(3, 80) = 0.62, *p* = .65. None of the institutional factors show significant associations with our dependent variable. In this model, Conscientiousness and impersonal causality orientation remain significant personality predictors as does self-rated ability to use technology for teaching remain a significant experience predictor for instructional resilience.

## Discussion

### RQ1:

What are the experiences of HE lecturers in maintaining teaching quality during ERT?

Our results suggest variation in terms of HE teacher’s experience during ERT. Although overall teaching quality took a significant hit, there is a large degree of heterogeneity, with some HE lecturers indicating a dramatic decline in teaching quality whereas others indicated hardly any or no difference at all. This degree of variance warrants explanation. We find that the degree to which teaching quality was affected is associated with the perceived challenge during ERT, which points toward the inherent difficulties of a sudden switch to fully-online instructional approaches. Although much relevant research is still emerging, by and large this aligns well with the broader narrative of significant challenges associated with the summer term of 2020 across the globe (Bozkurt et al., 2020; Crawford et al., 2020). This is also evidenced by the change in teaching load, which—although not overwhelmingly so—did noticeably increase. These results are similar to findings of Watermeyer et al. (2020) reporting work intensification in general during ERT.

At the same time, it seems that ERT has also provided an (involuntary) learning experience. HE lecturers report of substantial knowledge gains with respect to skills regarding teaching with technology due to their experiences of online and distance education during 2020. Although these are not ideal circumstances for professional development, HE lecturers seem to garnered experience in a variety of key learning design features, as more than half of respondents judged themselves able to implement presentation of content, feedback, assessment, and group learning. Interestingly, discussions during ERT was the most contested learning design features, despite it being somewhat of a staple of online learning and DE (Bernard et al., 2009). Possibly, HE lecturers are indeed able to design and facilitate discussions, yet they feel unable to attain the usual quality and benefits of in-person discussions. Further, we see a high and unambiguous self-rated ability to present content during ERT, hinting at the dominance of teacher-centered practices, what could be called the default mode of technology-enhanced learning (Margaryan et al., 2015; Tu, 2005). Some HE lecturers found themselves fully unable to provide more ambitious learning scenarios, as indicated by their free-text responses. This aligns well with the findings that HE lecturers “shift-down” instructionally during ERT, compared to face-to-face teaching (Rutherford et al., 2021) Tellingly, HE lecturers in our sample also echoed challenges that are traditionally associated with online-based forms of teaching and learning, like social aspects (Weidlich & Bastiaens, 2017; Kreijns et al., 2003), student engagement (Bond et al., 2020), and flexibility in learning design (Shute & Towle, 2003).

In terms of workplace challenges and supports during ERT, we found that social, organizational, and technical support covaried. From the viewpoint of HE lecturers, the interpretation suggests that if their institution displays high degrees of support in one area, it likely also provides other support. Negative correlations with workload suggest that high workload is associated with less satisfaction with these support factors. Yet, none of these workplace challenges and supports are associated with the perceived challenge during ERT. Apparently, HE lecturers perceived the challenges of ERT to be independent from workload and support factors. This appears to be a notable diversion from occupational psychology research showing that support structures and lower workload increase likelihood of good job performance (e.g. Talukder et al., 2018). One interpretation of this is that although institutional support factors are generally appreciated, they have limited value for the very specific and novel challenges posed by ERT leading again to a mostly individual challenge. This notion is supported by the open-ended questions relating to institutional aspects that made ERT particularly challenging. Although there are some thematic similarities (e.g. workload, support), responses point to more complex and nuanced issues at the interplay of individual and institution like disregard of increased workload, lack of transparency and communication, and quickly shifting requirements.

### RQ2:

Which factors contribute to experiencing instructional resilience during ERT in HE?

The results of our first model including only control variables of gender and age category point to only one significant effect. We found that HE lecturers in the oldest age category reported higher degrees of instructional resilience than those in the youngest category. At first glance, this may seem puzzling, as characteristics like technological readiness and flexibility are usually associated with younger individuals (Barak, 2018). On the other hand, flexibly adapting teaching while maintaining quality during a crisis is predicated on teaching expertise and, thus, teaching experience. As in teacher resilience, expertise and experience can be seen as resources on which more seasoned HE lecturers may draw when facing the challenges of ERT (Helker et al., 2018). However, these results should not be over-interpreted as this first model has relatively little predictive power. Moreover, the association of between age category and instructional resilience weakens once we introduce personality attributes.

In terms of personality attributes, we found a pattern of predictors that is robust across models. Firstly, and in contrast to expectations, our findings suggest that general psychological resilience does not predict instructional resilience, meaning that the general ability to adapt to and bounce back from crises does not significantly contribute to the more specific ability of HE lecturers to maintain teaching quality during ERT. We interpret these findings by referring to the relative domain-specificity of instructional resilience. With our measure of the construct we specifically tapped the ability to dynamically maintain teaching quality under the difficult circumstances of ERT. And although we suspected that general coping ability would play a role in this, it seems that the “instruction”-component of our focal construct plays a dominating role, which also aligns well with the finding that perceived ability regarding using technology for teaching purposes predicts instructional resilience (see next paragraph). However, the role of broad personality attributes should not be discounted. As we, along with other research (Watermeyer et al., 2020), note the increased workload and challenge of teaching during ERT, our results support the notion that there are individual differences that predispose HE lecturers toward tackling these challenges. As the strongest predictor, we find that conscientious HE lecturers displayed higher instructional resilience. Conscientiousness being associated with self-discipline and work ethos, this makes intuitive sense. However, another side of this dimension is carefulness, neatness and being systematic, which presumably played a lesser role in light of the sudden switch to ERT. Future research may want to assess this relationship more deeply by also modeling the sub-facets of Conscientiousness (Roberts et al., 2014) in relation to instructional resilience. Finally, impersonal causality orientation was a significant predictor of instructional resilience. As this aspect of personality is frequently associated with feeling ineffective and amotivated, it makes sense that HE lecturers scoring higher on this dimension would have managed worse during ERT. This aligns with findings that anxiety due to the Covid-19 pandemic is negatively associated with effectiveness during ERT (Alqabanni et al., 2020). Interestingly, and against expectations, the opposite of this, autonomous orientation, was not a significant positive predictor, implying an asymmetry with respect to risk versus protective factors, such that a positive motivational orientation did not help much toward instructional resilience but a negative motivational orientation indeed had negative consequences. This asymmetry is an interesting result and may provide an avenue for deeper understanding of instructional resilience in future investigations.

Instructional resilience was associated with prior relevant experience only on one account, self-reported ability to use technology for teaching. Although it is highly plausible that this type of experience would benefit HE lecturers in the switch to ERT, again highlighting the teaching-specificity of instructional resilience, it is also surprising that working in the field of TEL/DE and at an institution with online offerings do not function as protective factors here. Of course, it is entirely possible that HE lecturers working in the field of TEL/DE actually study and teach in-classroom technologies or work with other student populations than tertiary students. Also, working at an institution with online offerings does not necessarily imply that every sampled individual is associated with the offerings, nor that he/she is automatically proficient. However, with respect to organizational and social support, we did find some differences along these categories of relevant experience, it just appears that this has not translated into individual instructional resilience. Having previously engaged in some type of professional development with respect to TEL/DE was also a strong predictor, although not statistically significant due to high variance. This can be taken to mean there are benefits associated with this variable, although not for every individual. One possible explanation for this may that professional development was only effective if it was related to the specific challenges of ERT in HE, like fully online teaching and tertiary education. In sum, in terms of prior experience, we find the display of instructional resilience to be largely predicated on specific self-reported knowledge about using technology for teaching purposes, with no evidence that HE lecturers in the field of TEL/DE or at institutions with online offerings fared significantly better.

Finally, we find that neither organizational, social, and technical support nor workload significantly predicted instructional resilience. These are interesting results as they suggest limits to the degree to which institutions can play a role in promoting instructional resilience. Although support structures and a manageable workload are intrinsically valuable workplace characteristics, it seems that they have been rather inconsequential for instructional resilience during ERT. Also, the addition of these factors adds little in the way of predictive power of the model, as explained variance did not significantly increase with the inclusion of these variables.

## Limitations

One limitation lies with the sample size of our study. Although sufficient for most of our analyses, our main analysis, hierarchical regression, was only powered to detect a medium effect, *f*^2^ = .22, using G*Power (Faul et al., 2007), sensitivity analysis, alpha = .05, beta = 80%. This leads to wider confidence intervals around estimates and, thus, may have resulted in false negatives for some predictors.

Although we tried to sample HE lecturers from around the world and succeeded in receiving responses from a diversity of countries, overall our sample has a WEIRD bias (Henrich et al., 2010), with most responses from Germany, US, UK, and the Netherlands. These are countries that are technologically and economically well equipped, so that our results may not be applicable in countries that do not have these hallmarks.

Another limitation that may be considered in interpreting our findings is that our measure for instructional resilience was not independently validated. Ideally, scale validation is conducted on a separate dataset from the one on which inferences are made. However, given the timely nature of our research questions, this did not seem feasible. Our efforts at providing evidence for validity of this measure make us optimistic that we have worked with a scale of solid psychometric quality. Researchers may want to replicate these scale validation steps in their own sample before putting the scale to wide use.

Due to our desire to not overburden our respondents, we used short scales whenever possible (e.g. BFI-2-SX) and also shortened some existing scales. For example, the GCOS usually consists of 12 vignettes, of which we selected only those 6 that seemed most applicable for academia. In some cases, these small amounts of items have not served us well, for example the Openness dimension of the Big Five. As a result, we had to exclude this dimension. As this investigation was meant as a starting point and we attempted to sample a broad array of possible predictors, future research may do well to focus more specifically on a subset of predictors and investigate these more thoroughly.

On the other hand, despite our efforts at creating an economic questionnaire, a total of 75 items may still be perceived as a high load, especially given that there was no reward associated with completion. For this reason, we cannot rule out the possibility of fatigue or boredom in our respondents. In future research, careful piloting of the questionnaire may help ensure that respondents are not overburdened.

Finally, our investigation was very much post-hoc, relying on HE lecturers’ ability to introspect about past events. In our case, these events are months’ past, bringing about the possibility that our sample misremembered certain aspects of the experience. However, as the Covid-19 pandemic and the sudden switch to ERT appear to be once-in-a-lifetime events, we are optimistic that our participants are more likely to vividly recall these experiences than commonplace events. Still, upon interpreting our findings, readers should be aware of this possible issue.

## Conclusion

The Covid-19 pandemic has been a shock to the HE system. As the implications and consequences of the sudden switch ERT will likely be felt for years but are not yet fully understood, it is important to look deeply at how different stakeholders coped in 2020. In this investigation, we suggest that the extent to which education in HE was continually and successfully provided during ERT is the result of HE lecturers’ instructional resilience, their ability to maintain teaching quality by flexibly adapting in challenging circumstances. Our results show that ERT indeed led to a decrease of teaching quality as perceived by HE lecturers, likely due to the unique challenges posed by ERT. Yet, our results suggest that this decrease may be smaller than expected and quite heterogenous. HE lecturers made use of an array of learning design features to provide education during ERT and appear to have acquired knowledge and confidence towards online-based teaching as a result of this. Further, the ability to effectively adapt and modify teaching practices in this way appears to be associated with HE lecturers that are conscientious and already knowledgeable in using technology for learning, whereas HE lecturers that feel generally ineffective and are amotivated were less likely to display such instructional resilience. As an initial theoretical implication, this points to significant motivational and domain-knowledge dimensions of instructional resilience. Conversely, we can wager that institutional factors play a comparatively smaller role, placing the onus of instructional resilience squarely within the individuals’ abilities and dispositions. Further research may want to explore this more deeply, for example by utilizing established motivation frameworks like self-determination theory (Deci & Ryan, 2000) to understand how fostering basic needs like autonomy and competence may attenuate the potentially deleterious effects of impersonal causality orientation of HE lecturers.

Besides the theoretical implications presented above, the study also has practical implications: the role of relevant experience (specifically with regard to teaching with technology), personality, and motivation compared to the minor role of institutional factors suggests that institutional investments in training and skill development may pay off more than comparable investments into institutional support structures. Looking more closely at potential contents of such training, results point to the importance of developing HE lecturers’ skills related to group learning, discussion-oriented scenarios, as well as practice and application of knowledge to ensure a healthy mix of online-based learning and teaching practices. Yet, it may be premature to disregard the role of institutional factors as a result of this study. Given the generally high level of all support dimensions in the participants´ institutions within our sample, it seems reasonable to assume a decreasing utility of institutional factors above a certain threshold. Thus, a sample including more lecturers reporting unfavorable institutional factors may reveal a more prominent role of these factors in promoting instructional resilience.

Taken together, this study provides a rich perspective into HE lecturers’ experience and performance during ERT and introduces a novel construct that may be critical to understanding teaching in a crisis. Exploratory in nature, this research paints instructional resilience in rather broad strokes by laying out the landscape of possible predictors. As such, it should be considered as a starting point for broader as well as more focused future investigations. Broader, for example, by further validating and expanding these initial results with a larger and more diverse sample. More focused, as each of our three hypothesized predictor types, personality attributes, prior experience, and institutional factors could be investigated exclusively and in dedicated research endeavors, using more elaborate measures and data collection procedures, for example via mixed-methods.

It is readily apparent that ERT due to Covid19 has been a bane for students and educators across the world. Long-term, however, it may turn out to be a boon for the development of HE institutions, forcing their hand in developing institutional readiness for online-based methods of learning and teaching. Our investigation suggests that teaching during ERT, to some extent, has been carried out on the back of HE lecturers. As the pandemic carries on and HE institutions settle in for longer-term solutions, this situation surely must change by providing the infrastructure, professional development, and work environment such that quality online-based education can be attained in a consistent and durable fashion as well as relatively independent of the mode of delivery.

## Data Availability

Data and material for this study is available at the Open Science Framework, at [https://osf.io/s26rt/ and 10.17605/OSF.IO/S26RT].

## Appendix

### Instructional resilience

IR_1: My teaching quality did not suffer from the abrupt change to emergency remote teaching.

IR_2: Despite the switch to remote teaching, I was able to conduct my teaching with the usual quality.

IR_3: My teaching suffered from the emergency remote teaching of last semester. (-)

IR_4: I estimate that my students learned less in this past semester of emergency remote teaching than in the semesters prior. (-)

IR_5: Despite the challenges of the remote teaching semester, I was able to teach my students effectively.

IR_6: I was able to adapt my teaching to the conditions of remote teaching while upholding my usual standards of quality.

### General resilience

GR_1: I tend to bounce back quickly after hard times.

GR_2: I have a hard time making it through stressful times. (r)

GR_3: It does not take me long to recover from a stressful event.

GR_4: It is hard for me to snap back when something bad happens. (r)

GR_5: I usually come through difficult times with little trouble.

GR_6: I tend to take long to get over set-backs in my life. (r)

### TPACK before/after ERT

TPCK_1: I was/am able to use strategies that combine content, technologies, and teaching approaches in my teaching activities.

TPCK_2: I was/am able to choose technologies that enhance the content for a lesson.

TPCK_3: I was/am able to select technologies that enhance what I teach, how I teach, and what students learn.

TPCK_4: I was/am able to teach lessons that appropriately combine my teaching subject, technologies, and teaching approaches.

### Big Five

I am someone who…

- Tends to be quiet (E-)
- Is compassionate, has a soft heart (A)
- Tends to be disorganized (C-)
- Worries a lot (N)
- Is fascinated by art, music, or literature (O)
- Is dominant, acts as a leader (E)
- Is sometimes rude to others (A-)
- Has difficulties getting started on tasks (C-)
- Tends to feel depressed, blue (N)
- Has little interest in abstract ideas (O-)
- Is full of energy (E)
- Assumes the best about people (A)
- Is reliable, can always be counted on (C)
- Is emotionally stable, not easily upset (N-)
- Is original, comes up with new ideas (O)

### Causality orientation

You have been offered a new position in a company where you have worked for some time. The first question that is likely to come to mind is:

- What if I can't live up to the new responsibility? (I)
- I wonder if the new work will be interesting? (A)

You had a job interview several weeks ago. In the mail you received a form letter which states that the position has been filled. It is likely that you might think:

- I'm probably not good enough for the job. (I)
- Somehow they didn't see my qualifications as matching their needs. (A)

You have just received the results of a test you took, and you discovered that you did very poorly. Your initial reaction is likely to be:

- "I can't do anything right," and feel sad. (I)
- "I wonder how it is I did so poorly," and feel disappointed. (A)

You have been invited to a large party where you know very few people. As you look forward to the evening, you would likely expect that:

- You'll find some people with whom you can relate. (A)
- You'll probably feel somewhat isolated and unnoticed. (I)

Recently a position opened up at your place of work that could have meant a promotion for you. However, a person you work with was offered the job rather than you. In evaluating the situation, you're likely to think:

- You didn't really expect the job; you frequently get passed over. (I)
- You would probably take a look at factors in your own performance that led you to be passed over. (A)

You are embarking on a new career. The most important consideration is likely to be:

- Whether you can do the work without getting in over your head. (I)
- How interested you are in that kind of work. (A)

### Workload

W_1: The workload was is too heavy.

W_2: It sometimes seemed to me that my job requires me to do too many different things.

W_3: In this organisation you were expected to spend a lot of time learning things on your own.

W_4: There seemed to be too much work to get through here.

W_5: There was a lot of pressure on you as an employee here.

### Organizational support

OS_1: My organization values my contribution to its well-being.

OS_2: My organization fails to appreciate any extra effort from me. (R)

OS_3: My organization would ignore any complaint from me. (R)

OS_4: My organization really cares about my well-being.

OS_5: Even if I did the best job possible, my organization would fail to notice. (R)

OS_6: My organization cares about my general satisfaction at work.

OS_7: My organization shows very little concern for me. (R)

OS_8: My organization takes pride in my accomplishments at work.

### Technical support

TS_1: Technical support was readily available for inquiries.

TS_2: Technical supports provided clear answers and instructions to my inquiries.

TS_3: Technical support provided timely answers.

TS_4: Technical support demonstrated sufficient expertise to help address my problems.

### Social support

SS_1: If needed, can you get support and help with your work from your co-workers?

SS_2: If needed, can you get support and help with your work from your immediate superior?

SS_3: If needed, are your co-workers willing to listen to your work-related problems?

SS_4: If needed, is your immediate superior willing to listen to your work-related problems?

SS_5: If needed, can you talk with your friends about your work-related problems?

SS_6: If needed, can you talk with your spouse or any other close person about your work-related problems?
